# Collaborative Practice in the Management of Neuroendocrine Tumors

**Published:** 2016-04-01

**Authors:** Jennifer A. Chan, Robin Sommers

**Affiliations:** Dana-Farber Cancer Institute, Boston, Massachusetts

Management of neuroendocrine tumors (NETs) requires a multidisciplinary approach, as many modalities are useful in this malignancy. Somatostatin receptor analogues (SSAs) are a mainstay of treatment, both for ameliorating symptoms and slowing disease progression, but novel approaches are emerging. At 2015 JADPRO Live, Jennifer A. Chan, MD, MPH, and Robin Sommers, DNP, ANP-BC, AOCNP, both of Dana-Farber Cancer Institute, Boston, Massachusetts, brought attendees up to date on management strategies for NETs.

NETs arise from cells in the diffuse neuroendocrine system throughout the body. Traditionally, NETs have been divided into pancreatic NETs and NETs originating in the gastrointestinal (GI) tract, lungs, and other nonpancreatic sites, previously referred to as "carcinoid" tumors.

## COLLABORATIVE MODEL

Patients with NETs are best cared for by a multidisciplinary team that includes oncologists, nurses, surgeons, interventional radiologists, pharmacists, nuclear medicine specialists, and others. Dana-Farber has a collaborative practice model that integrates care between physicians and advanced practitioners (APs). The goals of this partnership are to improve patient care; increase clinical productivity; improve access for new patients; provide urgent care; provide coverage for physicians; and provide long-term care for cancer survivors, Dr. Sommers revealed.

APs, added Dr. Sommers, bring much to this collaborative model, which "offers patients the best of both worlds" and serves a growing practice well. "APs see our urgent-care patients, which frees up our physicians’ time, and cover for our physicians when they are out of town, with other MDs as backup," she continued.

## KEY FEATURES OF NETS

The key features of NETs are histology (well differentiated vs. poorly differentiated), grade, primary site, and functional vs. nonfunctional status. Histology and grade are associated with prognosis and influence management decisions, since poorly differentiated NETs are aggressive biologically and typically treated with platinum-based chemotherapy, Dr. Chan said.

The primary site—pancreatic vs. nonpancreatic sites—also reflects distinctions in terms of disease biology, genetics, and response to treatment. In the Dana-Farber database of almost 700 patients, median overall survival was 3.9 years for pancreatic NETs and 7.9 years for patients with small bowel (carcinoid) tumors (Ter-Minassian et al., 2013).

"Because response to treatment and survival vary by primary site, we have distinct treatment approaches and clinical trials based on pancreatic vs. nonpancreatic NETs," indicated Dr. Chan.

## FUNCTIONAL STATUS OF TUMORS

Functional NETs are associated with clinical symptoms related to the hormone that is secreted. "Carcinoid syndrome," which is related to secretion of hormones including serotonin, consists of symptoms such as flushing, telangiectasia, diarrhea, abdominal pain, wheezing or shortness of breath, palpitations, and valvular heart disease.

Approximately 30% of pancreatic neuroendocrine tumors are functional tumors. Symptoms experienced by patients with insulinoma include hypoglycemia; gastrinoma is associated with diarrhea and ulcers; VIPoma (a tumor secreting vasoactive intestinal peptide) causes secretory diarrhea and electrolyte abnormalities, Dr. Chan noted.

## WORKUP AND MANAGEMENT

Patient evaluation depends on the primary site and symptoms. Blood and urine tests include chromogranin A levels (a secreted peptide), 24-hour 5-HIAA (5-hydroxyindoleacetic acid, a product of serotonin), and other hormone or biochemical tests as clinically indicated. Imaging consists of cross-sectional CT (computed tomography) or MRI (magnetic resonance imaging), octreotide scans (for localizing the disease expressing the somatostatin receptor), and echocardiography to evaluate for valvular heart disease (particularly if there are symptoms of carcinoid syndrome).

Basic management principles are to control hormone secretion, control disease growth, and resect localized and limited metastases, Dr. Chan said.

## DISEASE CONTROL IN PANCREATIC NETS

For the treatment of pancreatic NETs, SSAs have been a mainstay for decades. The two US Food And Drug Administration (FDA)-approved SSAs—octreotide and lanreotide—bind to somatostatin receptors, which are highly expressed on NETs and can ameliorate hormone-mediated symptoms.

More recently, randomized data from two trials have shown that SSAs also have an antiproliferative effect and can improve disease control. In the PROMID study that included patients with midgut NET, median progression-free survival was 14.3 months with octreotide, vs. 6.0 months with placebo (Rinke et al., 2009). In the CLARINET trial, which enrolled patients with a broader range of NETs (pancreatic, GI, and others), lanreotide improved progression-free survival, the median of which was not reached in the lanreotide arm but was 18 months in the placebo arm (Caplin et al., 2014; see Figure).

**Figure 1:**
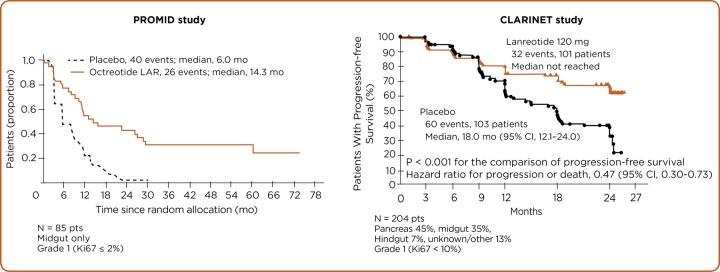
Octreotide and lanreotide for advanced neuroendocrine tumors. Adapted from Caplin et al. (2014); Rinke et al. (2009).

Side effects of the SSAs include disordered glucose regulation, hypo- and hyperglycemia, thyroid disorders, cardiovascular disorders, vitamin B_12_ deficiency, and gallbladder disease.

## OTHER AGENTS ACTIVE IN PANCREATIC NETS

Other agents active in advanced pancreatic NETs are pathway inhibitors: the mTOR (mechanistic target of rapamycin) inhibitor everolimus and inhibitors of the vascular endothelial growth factor (VEGF) pathway, including sunitinib.

Both everolimus and sunitinib have been shown to improve progression free-survival compared to placebo in patients with progressive pancreatic NET (Raymond et al., 2011; Yao et al., 2011). Although efficacy is similar, these agents differ in their toxicity profiles. Sunitinib is associated with hypertension, hand-foot syndrome, and fatigue. Everolimus produces more pneumonitis, hyperglycemia, and skin rash. These differences can drive treatment choice, Dr. Chan said.

Studies have examined whether dual pathway inhibition may be more effective. In the CALGB 80701 study, everolimus and octreotide plus bevacizumab, a monoclonal antibody targeting VEGF, improved progression-free survival compared to everolimus and octreotide (16.7 months vs. 14.0 months; hazard ratio = 0.80), but 81% of patients on the combination treatment experienced grade 3/4 toxicity (Kulke et al., 2015b).

Cytotoxic chemotherapy with alkylating agents, such as temozolomide or streptozocin, is also active in pancreatic NETs. There is interest in combining temozolomide with capecitabine, which is being investigated in ECOG 2211, a randomized study comparing this combination to single agent temozolomide (Kunz et al., 2015). Compared to somatostatin analogs and targeted agents, chemotherapy has been associated with higher radiographic response rates. "Response can translate into improvements in symptoms by reducing disease burden," pointed out Dr. Chan.

Although combinations may yield higher response rates and longer progression-free survival, they may be associated with increased toxicity, she acknowledged. "It raises the question of whether combinations are the right choice for patients who are also worried about quality of life," mentioned Dr. Chan. "We need to learn how to use them, in which patients, and for how long."

## SAFETY ISSUES WITH ORAL CHEMOTHERAPY

The use of oral agents for NETs raises safety concerns, according to Dr. Sommers. "We lose the system of checks and balances" with regard to refills, monitoring, drug interactions, and so forth, which was a component of infusional treatment, she added.

To enhance safety, Dr. Sommers and her colleagues developed a process requiring that all prescriptions be faxed to Dana-Farber’s oncology pharmacy. Within 24 hours of receiving the drug, the pharmacist calls the patient. The pharmacist also notifies the program nurses, who follow up with the patient in 72 hours to answer questions, resolve discrepancies, and confirm that the patient understands the treatment. The team calls the patient back within 2 weeks for a "quick toxicity check," to avoid the temptation for the patient to discontinue the drug when side effects arise.

## MANAGEMENT OF CARCINOID SYNDROME

The mainstay treatment for functional tumors is the use of SSAs, as they reduce hormone levels and accompanying symptoms related to hormone excess, but novel approaches are emerging.

One strategy is to target serotonin synthesis with telotristat etiprate, which inhibits tryptophan hydroxylase, the rate-limiting enzyme for serotonin production. In the phase III Telestar trial of patients with octreotide-refractory carcinoid, telotristat etiprate significantly reduced the frequency of bowel movements and reduced 5-HIAA levels (Kulke et al., 2015a).

## NETS OF THE GI TRACT, LUNG, AND THYMUS

Somatostatin analogs have been associated with improved progression-free survival in patients with GI NET and are currently being investigated in lung NET. In NETs associated with carcinoid syndrome, everolimus plus octreotide failed to meet its primary endpoint of demonstrating improved progression-free survival compared to placebo plus octreotide in the RADIANT-2 study (Pavel et al., 2011). However, everolimus was associated with progression-free survival benefit compared with placebo in the more recent RADIANT-4 trial of patients with progressive, nonfunctional tumors originating in the GI tract or lung (Yao et al., 2015). Median progression-free survival was 11.0 months, vs. 3.9 months (hazard ratio = 0.48; *p* < .00001), an encouraging result that led to its recent FDA approval for this indication.

Also promising is ^177^Lu-Dotatate, a peptide receptor radionuclide therapy that delivers tumoricidal doses of radiation to somatostatin receptor–positive tumors. The NETTER-1 study evaluated ^177^Lu-Dotatate in midgut NETs (functioning or not) that progressed on octreotide (Strosberg et al., 2015). Patients had a 79% reduction in the risk of disease progression (*p* < .0001), with median progression-free survival not yet reached with the radiopharmaceutical. The modality is available outside of the United States and hopefully will be approved here, Dr. Chan said.
